# Sensory Health and Dementia Care: Understanding and Addressing Unmet Needs in ADRD

**DOI:** 10.1093/geroni/igab046.323

**Published:** 2021-12-17

**Authors:** Carrie Nieman, Heather Whitson, Laura Gitlin

## Abstract

Sensory health in dementia stands at the intersection of two major public health challenges. Hearing and vision impairments are among the most common and disabling comorbidities in dementia and may worsen the trajectory of decline yet frequently go unrecognized and unaddressed. Improving sensory function may be an accessible and cost-effective nonpharmacological intervention to aid in the management of neuropsychiatric symptoms, improve quality of life for persons with dementia, and reduce burden for care partners. This symposium presents the latest evidence on the impact of sensory impairment in dementia and efforts to integrate sensory health into the care of persons with dementia. This symposium will cover emerging evidence of the impact of hearing loss and vision impairment on persons living with dementia, specifically around neuropsychiatric symptoms, disability, and cost. In moving toward solutions, we will discuss new approaches to provide vision and hearing care for persons with dementia in diverse settings, from audiology to specialized memory clinics to home-based care. This discussion will include findings from a systematic review of telehealth in dementia care, which highlights the limitations of existing literature on accounting for the sensory needs of persons with dementia and their care partners. Finally, we will share new international practice recommendations on vision and hearing impairment among persons living with dementia. The symposium highlights the large, yet often unrecognized, sensory health needs of persons with dementia and the multi-prong approach required to identify and support sensory health and, ultimately, healthy aging among persons with dementia.

